# Patients’ experiences of a behavioural intervention for migraine headache: a qualitative study

**DOI:** 10.1186/s10194-016-0601-5

**Published:** 2016-02-27

**Authors:** Myfanwy Morgan, Sian Cousins, Laura Middleton, Genevieve Warriner-Gallyer, Leone Ridsdale

**Affiliations:** King’s College London, Department of Primary Care and Public Health Sciences, Guy’s campus, London, SE1 1UL UK; King’s College London, Institute of Psychiatry, Psychology & Neuroscience, London, UK

**Keywords:** Headache, Migraine, Relaxation, Cognitive-behavioural therapy (CBT), Behavioural intervention, Self-management, Qualitative

## Abstract

**Background:**

Migraine headache has a high prevalence and a severe impact on personal, social and work life, forming a significant burden on patients, service providers and society. There is some evidence of the effectiveness of behavioural interventions to supplement drug therapy but a recognised need to identify an effective minimal contact approach to enhance access and provide a model for use in publicly funded health systems. This study uses in-depth interviews to examine patients’ experience and responses to a behavioural intervention with relaxation and CBT components delivered in three individual therapist sessions with follow-up.

**Methods:**

Qualitative study of 20 adults aged 18–75 years in London, UK, with clinically diagnosed migraine and at least four headache days per month. Semi-structured and tape recorded interviews were held post intervention based on a topic guide. Transcripts were coded and charted for all participants and analysed thematically.

**Results:**

The majority of participants cited the impacts of migraine and a desire for additional non-drug treatment as their main reasons for taking part and almost all completed the course. They valued contact with the therapist and almost all reported benefiting from the therapy. Post intervention they used those techniques they found most beneficial and implemented them flexibly in their daily life to reduce stress and risks of migraine or to respond to migraine. Relaxation training (deep breathing) was easily adopted and often used post intervention. The CBT components were mainly viewed positively but regarded as more challenging to learn and implement.

**Conclusions:**

Patients’ selectively identified and employed the techniques learned as ‘tools’ to assist in preventing and managing their migraines, with reported benefits supporting the development of minimal contact behavioural therapy to increase accessibility for adults with migraine headache and the conduct of a definitive trial.

## Background

Migraine headache is identified as the seventh most disabling condition worldwide [1] and has a severe impact on personal, social and work life as demonstrated by both quantitative assessments [2–4] and qualitative studies [5–7]. It is estimated that in the UK between 10 and 12 % of adults suffer with migraine, 6 % of men and 15–18 % of women [4]. Four percent of adults in the UK consult their family doctor for headache each year [8] and it is the most common reason for referral to neurologists [9]. Migraine is therefore regarded as imposing a high economic burden on patients, service providers, and society [10, 11].

A pharmaceutical approach to migraine is standard treatment and although beneficial has limitations including drug adherence, medication intolerance, patient preference and cost [12, 13] with patients therefore often seeking alternative remedies [6, 7, 14]. A solely pharmacological approach can also fail to take into account the social, cognitive and emotional factors associated with the onset, course and consequence of migraine, with psychological co-morbidities being common in headache patients [15, 16]. Psychologically mediated factors such as locus of control, self-efficacy and emotional states have been found to influence the course of headache by affecting perceived pain, migraine management and the overall impact of headache on related disability and quality of life [17–19]. This highlights the importance of a bio-psycho-social framework and non-drug behavioural interventions, including relaxation training, cognitive behavioural therapy (CBT) and biofeedback to help patients develop self-management skills and strategies to prevent and manage their headaches [20].

Behavioural interventions have most commonly been developed and tested in the United States [20]. However the US health systems heavy reliance on private insurance funding and likely cost barriers to this treatment reduces their accessibility. Although studies have also been undertaken in some European countries the forms of funding and systems of delivery again limit their generalisation to universal-access publically funded healthcare systems such as the National Health Service (NHS) in the UK which provides universal access in principle to all socio-economic groups. Responding to a situation where behavioural interventions have not been developed or tested for adults the UK’s National Institute for Health and Clinical Excellence (NICE) called in 2012 for research to evaluate the impact of psychological interventions on chronic headache disorders [13]

In response to this call a feasibility trial of a minimal contact behavioural therapy compared to standard medical treatment for patients with migraine was undertaken in the context of the UK’s public health system (21). The intervention involved a combined behavioural approach of deep breathing and progressive muscle relaxation techniques together with CBT therapy [18] The behavioural therapy was delivered by a CBT trained therapist over 5 weeks in three individual face to face sessions with two telephone calls to discuss progress. Participants were also given thought diaries to complete at home and asked to practise relaxation techniques with an accompanying CD for 15 min a day.

The feasibility trial showed adequate recruitment and 72 % attended all five treatment sessions [21]. However the documenting of treatment delivery needs to be accompanied by knowledge of the perceptions and enactment of therapies by participants to inform the development and evaluation of complex interventions [22]. The Medical Research Council (MRC) identified the contribution of qualitative research as integral to examining these issues [23]. This qualitative study therefore aimed to examine migraine patients’ prior expectations of the behavioural intervention, their experience of the differing components of the therapy, and views of the benefits and outcomes.

## Methods

Ethical approval was obtained from South East London Regional Ethics committee (10/H0805/79) and informed consent was obtained from all participants.

### Sample and recruitment

The qualitative sample was drawn from trial participants recruited from headache specialist clinics across London [21]. Patients were eligible for inclusion in the qualitative study if they had been assigned to the treatment group and completed a minimum of 8 weeks follow up from baseline assessment. Participants were contacted by the CBT therapist and asked if they would be willing to take part in an interview. Those who agreed were then contacted by a researcher.

### Interviews

Semi structured interviews were carried out by LM and GW-G and discussed with the other authors. A flexible interview guide was developed based on the literature and the aims of the trial together with three pilot interviews. This identified broad areas to be covered together with examples of suggested probes (see Box 1), although the interviewer was also free to follow up other issues that might arise and to probe and clarify responses. This approach led to discussions in which participants were able to identify and explain issues of importance to them rather than being limited by the narrower requirements of a structured questionnaire [24]. By the time 20 interviews had been undertaken no new themes were emerging indicating that saturation had been reached.

**Table 1:** Characteristics of respondents at baseline

Participant	Age	Gender	Employment	Headache frequency (per month baseline year)	Time between baseline & interview (no. weeks)
P1	30	F	Unemployed (Ill health)	23	23
P2	19	M	Unemployed	4	20
P3	56	F	Employed (Part time)	5	17
P4	45	M	Employed (Full time)	4	23
P5	51	F	Employed (Part time)	25	21
P6	22	F	Employed (Full time)	4	17
P7	43	M	Employed (Part time)	6	26
P8	48	F	Employed (Part time)	7	15
P9	27	F	Employed (Part time)	6	14
P10	54	F	Employed (Part time)	7	32
P11	28	F	Employed (Full time)	8	22
P12	70	M	Retired (Ill health)	24	37
P13	49	F	Employed (Full time)	7	41
P14	40	F	Employed (Full time)	8	50
P15	27	F	Employed (Full time)	6	22
P16	44	F	Self-employed	8	17
P17	47	F	Employed (Part time)	9	52
P18	63	F	Retired (Age)	5	15
P19	64	M	Retired (Age)	4	23
P20	25	M	Unemployed	28	15

Tape recorded interviews took place either at home or the research site according to the participants’ preference and lasted 30–80 min. The researcher’s position was described as someone separate to the trial and solely interested in the participants’ individual experiences of the intervention. Interviews were directly recorded on to a lap top with permission and transferred to a secure online file sharing website with recordings.

### Analysis

Recorded data were transcribed and entered into NVivo9, a qualitative software package. The original recordings were then deleted. Transcripts were initially read in full to gain an overall perspective of the data, followed by line by line open coding. The coded segments were grouped into themes which were then summarised and charted for each participant following a Framework approach [25]. Reliability and repeatability was enhanced by all authors reading a selection of transcripts, discussing codes and themes and reviewing the charted summaries, with subsequent discussion of interpretations.

## Results

### Participants

Twenty-five eligible participants were invited to take part and 21 agreed, although one did not attend due to illness. The majority of participants were female (*n* = 14) and three were of Black or mixed ethnicity (numbers 5, 9, 17). Ages ranged from 19 to 70 years, with a mean of 43 years. Fourteen were employed, with most in part-time employment (Table 1). All participants had established migraine and all had experienced migraine for many years apart from participant 4. The number of headache days reported over the month prior to baseline assessment ranged from 4 to 31 days.

### Prior perceptions and expectations of treatment

Participants had varying views and expectations of the therapy. Twelve described the main reason for their taking part as a desire to try a new approach after a long history of other treatments that were either not working for them or the side effects were not well tolerated, with those with more frequent seizures being particularly keen to try any alternatives. For example, Participant 1 who experienced 23 migraines a month explained her reason for wishing to participate:

“*Just to sort of try and see if there were other avenues, obviously I tried so many medications and they weren’t working, so I wanted to try something different, to see if it would help. You know, just trying anything*.” (Participant 1, 30 years)

#### Similarly participant five who reported 25 migraines a month observed

*“the fact that you get to talk about how you’re feeling or perhaps what you’ve used and looking at different ways of trying to manage the pain better, I think it is a useful tool as opposed to ‘ok you’ve got a migraine try these tablets, off you go, but if they don’t work come back and we’ll give you some more’.”* (Participant 5, 51 years)

However many with less frequent migraines were also keen to try a new approach to reduce the frequency and severity of their headache and improve their ability to cope. As participant 3 who experienced 5 migraines a month explained:

“*As I said to the therapist, if you told me that standing upside down in a bucket of baked beans singing the royal national anthem would work, I would do it, because they used to have such a hold on me.”* (Participant 3, 56 years)

In contrast to these positive responses seven participants expressed initial uncertainty about the value of the therapy in helping their headaches. Reasons included scepticism about the link between psychological mechanisms and migraine management, and how CBT would benefit them, as this participant observed:

*“I was quite cynical in thinking about the way you think would affect you know, bring on a medical condition if you like. I know in some cases people can be, you know psychologically, they can bring on and manifest things, but I wasn’t totally convinced that thinking in a different way would help, because I think you are who you are, and you think the way you think, so I was probably a bit cynical when I came on.”* (Participant 13, 49 years)

For a few participants what was particularly important for them was to gain a better knowledge and understanding of what caused their migraines. Moreover some participants who were sceptical of personal benefits expressed altruistic views in terms of the importance of participation in helping research and developing therapies for ‘fellow migraine sufferers.’

### Experience of the therapy

Overall just four participants described limited benefit from the behavioural therapy. Two of these people did not complete the sessions, with one giving personal circumstances as a reason and the second did not feel that the therapy suited him due to its emphasis on feelings and thoughts. The other participants all described some positive benefits despite initial scepticism in some cases.

The majority of participants described the relaxation component involving both deep breathing and progressive muscular relaxation (PMR) as beneficial. Relaxation techniques were mainly used as a means to try and prevent a migraine from coming on when they felt tense:

*“…if I get to a level where I’m really rushing around or I’m really stressed or just had a really busy day, I will just take that time and I identify that without even really thinking to identify it, if you know what I mean? It’s you know something that I sort of think, OK let’s do a ten seconds of breathing and just bring myself back down and that does help*.” (Participant 15, 27 years)

However some used relaxation techniques when the migraine had come on to reduce its intensity, although others found this too difficult. Relaxation techniques were thus generally seen as ‘a tool’ to be used if needed and particularly to reduce the onset of migraine. In particular participants commented positively on the flexibility of relaxation techniques, especially deep breathing which they were able to use in a variety of situations, such as when commuting and in the workplace *because “it’s not something that you have to be in a kind of complete silence away from public,”* whereas PMR was more difficult to make time for.

In contrast, three participants felt that the relaxation techniques were personally of limited use as they did not regard themselves as stressed or did not view this as a trigger factor and these participants therefore saw no value added in carrying out the exercises. However one of these participants had begun to question whether there might be a link with stress and the onset of migraine as she realised that her migraines had reduced after leaving her job.

For CBT the main challenge reported by over half the participants (*n* = 13) centred on identifying thoughts, emotions and behaviours and the links between them. Patients commented that this difficulty may stem from this previously being an autonomic, subconscious process and their lack of prior experience in analysing thoughts and behaviours closely. Some people also speculated that long held beliefs or behaviours surrounding their headaches may be harder to change compared with someone who had only recently started to suffer with headaches:

*“… Yeah but they are very linked and it’s very, very difficult to…unless you’re really seriously taking time to sit down and analyse it, I think it’s quite hard to consciously separate thoughts and feelings. Consciously I think it’s quite hard*.” (Participant 13,49 years)

Therapist contact and support were described as very important in changing perceptions to feeling hopeful about the ways in which the therapy could be effective:

‘*When [name of CBT nurse]explained, I was thinking I really want to be part of it (therapy sessions).....I’m going to know what it’s about and hopefully it will be good*.’ (Participant 10, 54 years).

Face-to-face therapist contact also helped to break down their thought processes and clarify connections between thoughts, emotions and behaviours, and they also appreciated the use of relevant examples. As this participant (16, 44 years) explained:

“*I think it’s a really hard thing to do and to do it on your own would be nearly impossible.”*

Overall over half of the participants (*n* = 13) reported the CBT component of the treatment had benefited them in some way, particularly by reducing stress and improving general levels of positivity.

*“CBT has given me an added tool to look at certain situations that I might think are stressful or how can I resolve it or whatever, and it’s given me that extra tool in how to process that through.”* (Participant 10, 54 years)

CBT was also found to be beneficial for a few people in identifying and managing triggers, as well as improving coping strategies for when a headache occurred, with one participant reporting a reduction in the intensity of her headaches after implementing these techniques.

*“Sometimes when I have that migraine, you do get these really like ‘churney’ thoughts that are kind of pain related but you often get like…if you’re anxious about something as well…you do get like, the pain and the anxiety do get kind of mixed up, so I think having the cognitive behavioural therapy kind of helps for those moments*.” (Participant 11,28 years)

Some participants however found difficulty undertaking the CBT component or described its limited usefulness, explaining that their migraines did not have an emotional component and examining their thoughts and emotions would therefore not help, while one participant thought that it could lead her to overanalyse events.

### Content, delivery and changes

Participants were generally happy with the number of face-to face sessions which was not regarded as overly demanding, especially as many lived close to the hospital. They were also very positive about the helpfulness and approach of the therapist. However some found the thought-challenging and alternative thinking particularly difficult and would have welcomed the opportunity to spend more time on this component to increase their confidence. This was important not only for the opportunity of being able to work through individualised examples, but also for participants’ ability to ‘open up’. Some said they would be less likely to do this over the phone although the check-in phone calls between sessions were regarded as helpful in having someone knowledgeable and empathetic to talk to. The manual was also well received and regarded as clear and easy to understand.

## Discussion

Traditionally qualitative studies of people with migraine mainly focused on facilitating the physicians’ consultations and use of acute medicines, whereas a small number of recent studies have examined patients’ experiences and their management and decision making for headache in their daily life [5–7, 14, 26]. The current study extended this and employed qualitative methods to elicit patients’ experience and decision-making in relation to a behavioural intervention for migraine headache. This process evaluation, recommended in the context of complex intervention trials [22, 23] has not previously been done by trialists in this area.

Participants’ accounts in the present study confirmed previous reports of migraine patients’ strong desire to find ways of reducing the frequency and severity of their headache and use of a range of non-pharmacological approaches. This desire for alternative ways of preventing and responding to migraine formed an important reason for initial uptake of the behavioural intervention, and all but two participants attended all five sessions. Although initial uptake may reflect a search for new ways of managing their condition together with notions of altruism, continuation appeared to be linked with feelings of personal gain with the majority of patients finding some benefit from the therapy. This occurred for patients who varied in their frequency of migraine and length of headache history who shared a similar concern to find ways of reducing the impact of migraine on their lives.

The positive effects of the therapy occurred in a variety of ways, including the use of techniques as tools for everyday stress-management, trigger-management and increasing levels of positivity and feelings of control. Most participants found the deep breathing technique introduced in relaxation training was easy to implement, flexible to use, and beneficial in reducing stress, whereas the main challenge centred on the CBT component of the therapy in identifying thoughts, emotions and behaviours and the links between them. Face-to-face therapist contact was valuable in enabling participants to ‘open up’ about their emotions and headache related thoughts in the context of a collaborative relationship to help overcome challenges and to provide information and reassurance, as well as guidance about self-management.

Post intervention the participants often took the components they found useful from the treatment and implemented them in an individual way as ‘tools’ to either prevent and respond to triggers or manage their migraine. Responses were thus individualised with participants forming active decision-makers, identifying and enacting those approaches they perceived as most appropriate and beneficial to them in terms of their own beliefs and circumstances. This reflects previous descriptions of migraine patient’s active management of their condition, with newly gathered information influencing their next decision, behaviour and headache severity [5, 6] and supports the importance of a patient centred approach with migraine management tailored to individuals and access to different behavioural techniques [27, 28]. Moreover there is evidence that patients’ perceived self-efficacy and bolstering confidence in their ability to take actions to prevent or manage headaches is inversely associated with anxiety and related disability [18].

Qualitative studies take an in-depth approach and focus on small samples with the aim of achieving conceptual rather than statistical generalisation [24]. This is enhanced by the correspondence in findings across studies based on different areas and populations. The present study both supports the earlier notion of patients as actively involved in seeking additions to drug therapy to reduce the impacts of migraine on their lives [6, 7, 26] and also provides new evidence of the acceptability and benefits of minimal contact group therapy for migraine headache. However a number of aspects require further consideration. These include the recognised needs of some groups, including older people and ethnic minorities, for greater support to make the transition from pre-contemplation to contemplation and uptake with a need for monitoring [27]. It is also known that behavioural interventions are highly dependent on the skills and training of the therapist [29] and achieving fidelity in delivery [30]. Our findings also indicate that participants highly valued the therapist contact, especially the face-to-face sessions, which has also previously been found to be important from a patients’ perspective [27]. This has implications for the delivery of treatment, given the rise of low-cost internet-delivered treatment options [31–33]. Moreover whereas participants found the deep breathing technique introduced in relaxation training together with the audio guide as easy to implement, flexible to use, and beneficial in reducing stress, more sessions with a therapist or the provision of home based audio materials may be required to increase the implementation of CBT techniques post intervention.

## Conclusions

Participants’ accounts of their experiences of the behavioural intervention and use of the ‘tools’ post intervention supports this minimal contact multi-component group therapy as a model that enables participants to select the technique(s) that they believe will meet their specific needs at a given point or in a given environment or situation. It also has potential to enhance access to these treatments in publicly funded health systems and more generally addresses a key question of how behavioural interventions can be made more accessible and practical for adults with primary headache [34] and provides support for conducting a definitive trial.

## Box 1: Extract from topic guide with illustrative questions

**Table:** 

**Prior perceptions and expectations of treatment**
Did you have any particular reasons for agreeing to take part in the research?
Did you have any ideas about how this therapy may or may not help you before you started it?
**Cognitive behavioural therapy (CBT)**
How did you find the CBT sessions? Did you enjoy them?
Do you think they benefitted you in any way? How?
Did you find it easy to distinguish your thought from your feelings?
Do you think this had an impact on your migraines?
**Relaxation**
How did you find the relaxation therapy?
What did you like about the slow breathing exercise? Was there anything you didn’t like about it?
Did you notice any benefits to your migraines? What were they?
**Assessment of intervention**
What did you think about the number of group sessions? Too many, about right, too few?
Were the phone calls helpful? In what way?
What did you think of the manual? How did you use it?
Have you continued to use any of the techniques taught in the group sessions?
